# Too Dry for Primary Adrenal Insufficiency (PAI): PAI Masked by Volume Depletion

**DOI:** 10.7759/cureus.61018

**Published:** 2024-05-24

**Authors:** Jasdeep S Bathla, Anirudh Chitale, Shahzana Shahzad, Mohamed Elbathani, Nagaratna Sarvadevabatla

**Affiliations:** 1 Internal Medicine, Wayne State University/Detroit Medical Center, Detroit, USA; 2 Internal Medicine, Wayne State University School of Medicine, Detroit, USA

**Keywords:** falls, dizziness, dehydration, hypovolemia, acute hyponatremia, adrenal insuficciency, primary adrenal insufficiency, adrenal

## Abstract

Adrenal insufficiency (AI) is a rare but potentially life-threatening endocrine disorder characterized by inadequate production or impaired response to adrenal hormones. Symptoms may range from acute emergent crisis presenting as hemodynamic shock or may be more chronic in presentation with a gradual onset of nonspecific symptoms. These vague symptoms are often accompanied by typical laboratory findings, such as hyponatremia, hypotension, and hyperkalemia, and point toward a diagnosis of chronic AI. In this case presentation, we demonstrate chronic AI presenting with severe hyponatremia, which was revealed after return to an euvolemic baseline. Because of an insidious presentation, AI can be both an incidental finding and easily missed. This case highlights the importance of evaluating suspected cases of AI at a baseline metabolic and hemodynamic state, including volume status. High clinical suspicion is warranted in these patients to avoid potential emergent adrenal crisis and to provide appropriate replacement therapy once etiology is established.

## Introduction

Adrenal insufficiency (AI) can manifest acutely as an adrenal crisis or subacutely over time as a part of a nonspecific collection of symptoms. Initial signs of an adrenal crisis typically include hemodynamic shock, but nonemergent, chronic cases can include nondescript symptomatology including weakness, fatigue, nausea, and abdominal pain. Depending on the level of the hypothalamus-pituitary-adrenal (HPA) axis that is affected, symptoms and treatment may vary. As such, the veiled presentation of this condition can often be hidden by confounding hemodynamic and metabolic factors. Here, we present a case of AI diagnosed following resuscitation to euvolemic status.

## Case presentation

A 66-year-old female with a history of longstanding hypothyroidism of unknown etiology presented to our facility following an acute, mechanical fall at home. Medical history was notable for right-sided motor deficits due to a prior stroke, a left arm amputation suffered during childhood due to a deep vein thrombosis, and a myocardial infarction a few years ago. Over the last few months, she had experienced dizziness described as a room-spinning sensation that manifested as a feeling of off-balance and resulted in recurrent, mechanical, non-traumatic falls. She endorsed generalized weakness and fatigue along with poor oral intake but denied prodromal symptoms of syncope, including headaches, vision changes, sweating, or palpitations. 

On admission, she was normotensive with a blood pressure of 118/76 mmHg and negative orthostatic vital signs. The physical exam revealed sharp mental acuity with no new focal or lateralizing neurological deficits, and skin examination showed poor skin turgor with no hyperpigmented areas. Initial CT imaging of the head showed generalized volume loss and encephalomalacia in the left posterior frontal and parietal regions, consistent with a prior stroke. The cardiac workup returned negative for EKG changes, and a 2D echocardiogram revealed stable anteroseptal and apical akinesis. Initial labs were significant for severe electrolyte disturbances (hyponatremia, hypokalemia, and hypochloremia) and a glucose reading on the lower end of normal (Table 1). After confirming the results with our laboratory and giving a 500 mL bolus of normal saline, repeat labs were obtained, which showed mild improvement (Table 1). Medications were reviewed for a possible pharmacologic etiology of her electrolyte derangements and previous lab data from five years ago showed a normal sodium level of 135 mmol/L. The patient was admitted to the medical intensive care unit for monitoring of her severe hyponatremia.

**Table 1:** Initial and repeat values of measured serum electrolytes mmol/L = millimole per liter, mg/dL = milligram per deciliter

Lab	Initial value (a)	Repeat value (b)	Facility reference value
Sodium (Na)	84 mmol/L	116 mmol/L	136-145 mmol/L
Potassium (K)	3.3 mmol/L	3.8 mmol/L	3.5-5.1 mmol/L
Chloride (Cl)	62 mmol/L	83 mmol/L	98-107 mmol/L
Glucose	73 mg/dL	72 mg/dL	75-105 mg/dL
Blood urea nitrogen (BUN)	4 mg/dL	4 mg/dL	7-25 mg/dL

An initial serum osmolality was calculated to be low, with low urine sodium and a high urine osmolality pointing toward a hypotonic and hypovolemic etiology for hyponatremia (Table 2). Per nephrology recommendations, the patient was fluid resuscitated with normal saline boluses and maintained a strict free water restriction, after which serum sodium levels improved to 130 mmol/L. At this time, the patient began to develop worsening watery diarrhea, and sodium levels began to decline once again with hyponatremia of 120 mmol/L and hyperkalemia of 5.2 mmol/L. A repeat workup in a euvolemic state was obtained for refractory hypotonic hyponatremia, which now showed low serum osmolality, with high urine sodium and high urine osmolality (Table 2). Thyroid function was checked and thyroid stimulating hormone levels returned within normal limits, ensuring adequate dosing and administration of levothyroxine at home. Given renal salt wasting along with ongoing diarrhea, generalized fatigue with poor oral intake, and episodes of relative hypotension, concern for AI was raised. 

**Table 2:** Initial and repeat values of serum and urine labs for hyponatremia mOsm/kg = milliosmole per kilogram, mmol/L = millimole per liter

Lab	Initial value	Repeat value	Facility reference value
Serum osmolality	257 mOsm/kg	257 mOsm/kg	275-305 mOsm/kg
Urine sodium	17 mmol/L	136 mmol/L	20 mmol/L
Urine osmolality	404 mOsm/kg	558 mOsm/kg	50-1200 mOsm/kg

Early morning random cortisol level returned significantly low at <0.4 ug/dl. Due to the complicated clinical picture thus far, our endocrinology specialists recommended obtaining a cosyntropin stimulation test, rather than a direct ACTH measurement to avoid time delay with a send-out ACTH lab. Following stimulation testing, cortisol levels showed a mild increase to 2.1 ug/dl, still well below our facility cut-off minimum of 6.7 ug/dl. These results were suggestive of a primary adrenal insufficiency (PAI), and the patient was started on replacement therapy with hydrocortisone 20 mg in the mornings and 10 mg in the evenings along with fludrocortisone 0.1 mg daily. Sodium levels were corrected appropriately from 121 mmol/L to 133 mmol/L, and replacement dosing was adjusted to hydrocortisone 10 mg in the morning and 5 mg in the evening. Ultimately, the ACTH level returned high, confirming our diagnosis of PAI. To investigate etiology further, we obtained a CT of the abdomen and pelvis, which showed atrophy of the bilateral adrenal glands without any masses, in comparison to prior CT imaging in 2017 obtained for abdominal pain, which showed normal bilateral adrenal glands. Antibody testing for 21-α-hydroxylase antibodies returned negative. She was scheduled for a follow-up at our outpatient endocrinology clinic for further evaluation and continued care; however, she has unfortunately not been seen in our outpatient clinic.

## Discussion

Upon initial presentation, our patient endorsed a vague array of symptoms including weakness, fatigue, and dizziness. Her history of recurrent mechanical falls was attributed to a combination of her physical disabilities, which included a prior stroke with right-sided motor deficits and a left arm amputation, along with hyponatremia of undetermined duration that was possibly manifesting as recurrent spells of dizziness. The hyponatremia was believed to be due to volume depletion from poor oral intake and diarrhea as she had been having trouble taking care of herself independently. This was further supported by a positive response to fluid resuscitation and free water restriction, which helped normalize sodium levels as an euvolemic state was achieved. Following normalization, she demonstrated episodes of hypotension with refractory hyponatremia concerning renal salt wasting along with a high normal potassium, low normal blood sugar, and low blood pressure pointing toward an AI. 

The clinical presentation of AI can be divided into acute crisis versus chronic insufficiency. Acute adrenal crises are medical emergencies, often presenting in the setting of hemodynamic shock from an acute infection, adrenal infarction, or other stressors with constitutional symptoms, including malaise, fever, abdominal pain, nausea, vomiting, headache, and confusion [1,2]. Treatment includes supportive care with fluid resuscitation along with glucocorticoid replacement. In our patient, stool infection with *Clostridium dificile *was considered given her initial presentation, but her diarrhea had been ongoing for a few weeks and had shown complete resolution at times, making it difficult to even obtain a sample for testing. Our patient never displayed any signs of systemic infection or hemorrhage, and CT imaging did not reveal any masses that may have been interpreted as malignant or metastatic. Chronic insufficiency may present to an equal or lesser degree, with potential clinical findings including hyperpigmentation, postural hypotension, hyponatremia, hyperkalemia, and acidosis [2]. Upon presentation, our patient only exhibited hyponatremia while endorsing a nonspecific array of symptoms including weakness, dizziness, and fatigue, making it difficult to narrow down to an endocrine etiology. Hypovolemia, such as in our patient, may develop in an acute adrenal crisis due to cytokine release, which increases vascular permeability and fluid shifting. Although our patient did not present in an overt crisis, the presence of hypovolemia may suggest a milder form of acute crisis. 

Diagnosis of AI involves low serum cortisol concentrations, specifically during morning measurement when there is an expected, endogenous spike in hormone levels. Although a severely low AM cortisol with correlating symptoms may be sufficient to confirm AI, an adrenocorticotropic hormone (ACTH) level helps to distinguish a primary from a secondary or tertiary cause of AI [2,3]. A high ACTH signifies an issue with the adrenal glands themselves due to loss of direct negative feedback from a lack of cortisol production, whereas low ACTH levels demonstrate central insufficiency involving the hypothalamic-pituitary-adrenal axis. In complex clinical cases such as this one, it may be reasonable to proceed directly with a cosyntropin-stimulation test rather than ACTH measurement to avoid indeterminate results [3]. Cosyntropin, a synthetic form of hormonal ACTH, works to stimulate cortisol production directly from the adrenal glands. A low cortisol level after stimulation with standard high-dose cosyntropin (positive test) points toward AI due to the inability of the adrenal glands to produce cortisol; however, an ACTH level is then required to differentiate between primary or central causes [4]. 

In our patient, ACTH levels returned high confirming our diagnosis of primary AI. Adrenal imaging showed gland atrophy worsened from previous imaging, which was consistent with our diagnosis. Antibodies against adrenal hormones, most commonly 21-α-hydroxylase, as well as HLA class II typing, result in both humoral and cellular immune responses against the gland. Autoimmune adrenalitis, otherwise known as Addison’s disease, is the most common form of primary AI, and the 21-α-hydroxylase antibodies are present in up to 86% of cases, which show a mild hereditary pattern [5,6]. A large portion of autoimmune AI is categorized as idiopathic and may rarely present with negative antibody testing and normal CT imaging [7]. Alternatively, exogenous use of corticosteroids is the most common cause of central AI including secondary and tertiary etiologies [8]. 

Ultimately, treatment consists of addressing the underlying cause, if there is one, and steroid replacement depending on the level of defect and area of the adrenal gland that is involved [3]. Typical treatment includes glucocorticoids to synthetically replicate hormone function and the addition of a mineralocorticoid for primary causes.

## Conclusions

AI, when presenting subacutely, can be difficult to diagnose due to a gradual onset of generalized, nonspecific symptoms. In this instance, as described above, AI may be hidden by hemodynamic factors, such as fluid and electrolyte imbalances. Diagnosis is made by analyzing the hypothalamus-pituitary-adrenal axis and the hormones involved with this delicate system. Treatment ultimately revolves around managing the triggering events and usually will require steroid replacement hormones. This case emphasized the high clinical suspicion required for patients who present non-emergently, as symptoms may fluctuate and may not be discernible until returned to a balanced metabolic state.
